# Draft Genome Sequence of Proteus mirabilis UMB1310, Isolated from the Female Urinary Tract

**DOI:** 10.1128/MRA.00390-20

**Published:** 2020-05-14

**Authors:** Olivia Allen, Taylor Miller-Ensminger, Adelina Voukadinova, Alan J. Wolfe, Catherine Putonti

**Affiliations:** a Department of Biology, Loyola University Chicago, Chicago, Illinois, USA; b Bioinformatics Program, Loyola University Chicago, Chicago, Illinois, USA; c Department of Microbiology and Immunology, Stritch School of Medicine, Loyola University Chicago, Maywood, Illinois, USA; d Department of Computer Science, Loyola University Chicago, Chicago, Illinois, USA; University of Maryland School of Medicine

## Abstract

Proteus mirabilis is a Gram-negative bacterium that is linked to common complications within the urinary tract. Here, we present the draft genome for P. mirabilis UMB1310, which was isolated from the urine of a woman with a urinary tract infection.

## ANNOUNCEMENT

Proteus mirabilis is a motile bacterium often found within the gastrointestinal and urinary tracts (1). Due to its ability to overtake other bacteria through a coordinated movement called swarming, P. mirabilis is commonly associated with urinary tract infections (UTIs), bladder inflammation, urinary stones, and kidney infections (1, 2). P. mirabilis causes 12% of complicated UTIs, 15% of long-term UTIs, and 1 to 10% of all UTIs (3). This is related to its production of urease, which increases pH and frequently causes the formation of urinary stones and catheter blockages (4). Here, we present the genome for a P. mirabilis strain isolated from a catheterized urine sample obtained from a female with a UTI.

P. mirabilis UMB1310 was collected as part of a prior institutional review board (IRB)-approved study (5) using the expanded quantitative urine culture (EQUC) method (6). Briefly, the urine sample was obtained from a patient at Loyola University Medical Center’s Female Pelvic Medicine and Reconstructive Surgery Center (Maywood, IL, USA) between June 2014 and August 2015. One hundred microliters of urine was spread onto a Columbia nalidixic acid (CNA) agar plate and incubated at 35°C in a 5% CO_2_ environment for 24 h. Each distinct colony morphology on this plate was subcultured to obtain a pure culture for microbial identification. The genus and species were identified through matrix-assisted laser desorption ionization–time of flight (MALDI-TOF) mass spectrometry, and the sample was stored at −80°C. The sample was streaked onto a CNA agar plate using the four-quadrant streaking method and incubated at 35°C in a 5% CO_2_ environment for 24 h. After incubation, a single colony was picked, placed into 1 ml of lysogeny broth (LB), and incubated overnight. DNA was extracted using the Qiagen DNeasy blood and tissue kit following the protocol for Gram-positive extractions with the following exceptions: 230 μl of lysis buffer (180 μl of 20 mM Tris-Cl, 2 mM sodium EDTA, and 1.2% Triton X-100 and 50 μl of lysozyme) in step 2 and an incubation time of 10 min in step 5. The purified DNA was quantified using a Qubit fluorometer and sequenced at the University of Pittsburgh Microbial Genomic Sequencing Center. An Illumina tagmentation enzyme was used to enzymatically fragment the DNA. Indices were attached via PCR and sequenced using the Illumina NextSeq 550 platform, producing 2,370,675 pairs of 150-bp reads. Unless specified, default parameters were used for all software tools. The raw reads were trimmed using Sickle v1.33 (https://github.com/najoshi/sickle) and then assembled using SPAdes v3.13.0 with the “only-assembler” option for k values of 55, 77, 99, and 127 (7). The genome coverage was calculated using BBMap v38.47 (https://sourceforge.net/projects/bbmap/). Annotation of the genome was performed using PATRIC (8, 9), as well as the NCBI Prokaryotic Genome Annotation Pipeline (PGAP) v4.11 (10). The latter is published with the publicly available genome assembly.

The P. mirabilis UMB1310 draft genome is 3,945,930 bp long with a coverage of 150× and a GC content of 38.65%. It is composed of 95 contigs with an *N*_50_ score of 83,782 bp. The PGAP annotation identified 3,532 protein-coding genes, 78 tRNAs, and 6, 3, and 4 5S, 16S, and 23S rRNA sequences, respectively. The PATRIC annotation identified 50 coding regions associated with antibiotic resistance, including Tet(J), associated with tetracycline resistance. The genome does not contain the CRISPR/Cas system (11). To identify potential phages, we used PHASTER (12), which predicted 6 phages. However, only 1 was predicted to be intact, exhibiting the greatest sequence homology to *Enterobacteria* phage mEp460 (GenBank accession no. NC_019716; 25% query coverage and 67.57% sequence identity).

Further research on the P. mirabilis genome can aid future discovery and understanding of the prevalence of this pathogenic bacterium in the urinary tract.

### Data availability.

This whole-genome shotgun project has been deposited in GenBank under accession no. JAAUWF000000000. The version described in this paper is the first version, JAAUWF010000000. The raw sequencing reads have been deposited in the SRA under accession no. SRR11441013.
